# Enhanced self-supervised monocular depth estimation with self-attention and joint depth-pose loss for laparoscopic images

**DOI:** 10.1007/s11548-025-03332-1

**Published:** 2025-02-28

**Authors:** Wenda Li, Yuichiro Hayashi, Masahiro Oda, Takayuki Kitasaka, Kazunari Misawa, Kensaku Mori

**Affiliations:** 1 Graduate School of Informatics, Nagoya University, Furou-cho, Chikusa-ku, Nagoya, Aichi 464-8601 Japan; 2 Information Technology Center, Nagoya University, Furou-cho, Chikusa-ku, Nagoya, Aichi 464-8601 Japan; 3 Faculty of Information Science, Aichi Institute of Technology, Toyota, Aichi Japan; 4 Aichi Cancer Center Hospital, Nagoya, Aichi Japan; 5 Research Center of Medical Bigdata, National Institute of Informatics, Tokyo, Japan

**Keywords:** Depth estimation, Joint loss function, Self-supervised learning, Laparoscopic images

## Abstract

**Purpose:**

Depth estimation is a powerful tool for navigation in laparoscopic surgery. Previous methods utilize predicted depth maps and the relative poses of the camera to accomplish self-supervised depth estimation. However, the smooth surfaces of organs with textureless regions and the laparoscope’s complex rotations make depth and pose estimation difficult in laparoscopic scenes. Therefore, we propose a novel and effective self-supervised monocular depth estimation method with self-attention-guided pose estimation and a joint depth-pose loss function for laparoscopic images.

**Methods:**

We extract feature maps and calculate the minimum re-projection error as a feature-metric loss to establish constraints based on feature maps with more meaningful representations. Moreover, we introduce the self-attention block in the pose estimation network to predict rotations and translations of the relative poses. In addition, we minimize the difference between predicted relative poses as the pose loss. We combine all of the losses as a joint depth-pose loss.

**Results:**

The proposed method is extensively evaluated using SCARED and Hamlyn datasets. Quantitative results show that the proposed method achieves improvements of about 18.07$\%$ and 14.00$\%$ in the absolute relative error when combining all of the proposed components for depth estimation on SCARED and Hamlyn datasets. The qualitative results show that the proposed method produces smooth depth maps with low error in various laparoscopic scenes. The proposed method also exhibits a trade-off between computational efficiency and performance.

**Conclusion:**

This study considers the characteristics of laparoscopic datasets and presents a simple yet effective self-supervised monocular depth estimation. We propose a joint depth-pose loss function based on the extracted feature for depth estimation on laparoscopic images guided by a self-attention block. The experimental results prove that all of the proposed components contribute to the proposed method. Furthermore, the proposed method strikes an efficient balance between computational efficiency and performance.

## Introduction

During laparoscopic surgery, the narrow field of view (FoV) for surgical visualization is the main limitation facing operators [1]. Depth information is crucial in computer-assisted surgery systems. In robotic-assisted procedures, depth values support precise mapping of the surgical landscape and monitoring of surgical instrument movements in a wide 3D view [2]. Moreover, depth data are pivotal for virtual and augmented reality, permitting the creation of 3D models and facilitating the training of surgical technique [3].

Recently, self-supervised learning has been applied to the depth estimation task [4]. Zhou et al. [5] first proposed a depth-pose self-supervised learning framework by predicting the relative pose between adjacent images based on structure from motion (SfM) techniques. Godard et al. [4] improved this self-supervised learning framework by introducing novel auto-masking with a minimum re-projection loss. Lyu et al. [6] redesigned the skip connection and proposed a fuse feature module to improve performance on high-resolution datasets. Zhao et al. [7] leveraged COLMAP to refine poses for the depth estimation task on static scene datasets. Saunders et al. [8] proposed novel data augmentation with a pseudo-supervised loss for depth estimation on autonomous driving datasets. Wang et al. [9] introduced orthogonal planes to guide the depth estimation based on an augmented self-distillation loss on autonomous driving datasets. Due to the annotated datasets obtained from depth cameras and other hardware, these methods mainly focus on autonomous driving and static scene datasets [10, 11].

As one of the early approaches of depth estimation for laparoscopic images, many works have focused on providing large datasets. Ye et al. [12] provided laparoscopic stereo image datasets without depth values as ground truth. Allan et al. [13] organized a challenge by collecting an annotated dataset named SCARED using porcine cadavers. Recasens et al. [14] processed Hamlyn datasets and annotated videos to create useful datasets for depth estimation.

**Fig. 1:**
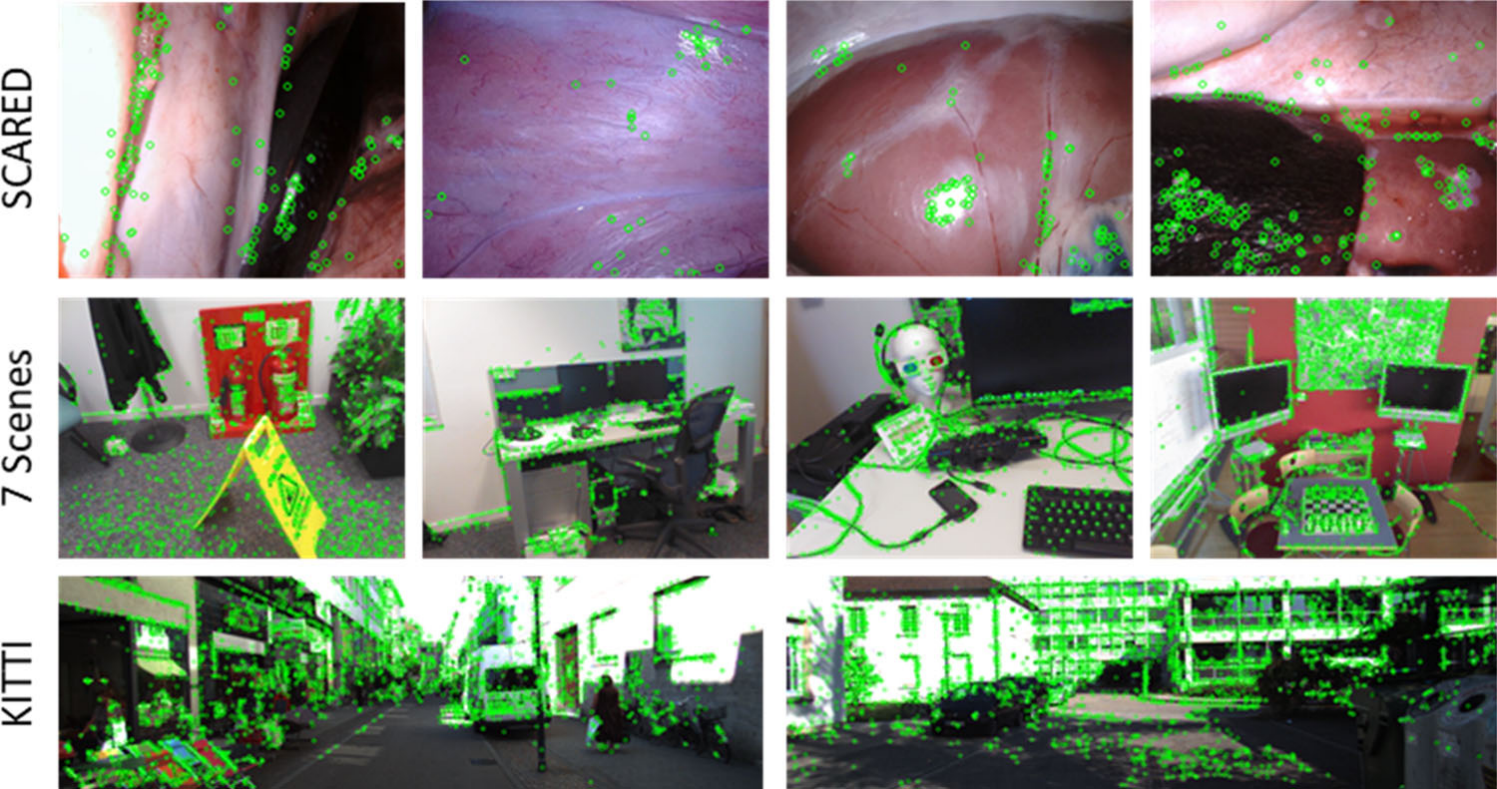
Illustration of key points on RGB images with different scenes. From top to bottom by row, the RGB images are from SCARED datasets [13], 7 scenes datasets [10], and KITTI datasets [11]. The green circles present the key points detected by scale-invariant feature transform (SIFT) [15]. These key points are fewer in laparoscopic scenes and the images lack rich texture compared to other scenes

Based on these datasets, researchers started applying self-supervised monocular depth estimation on laparoscopic scenes. In pioneering research, Ye et al. [12] introduced the Siamese network to estimate disparity maps. Li et al. [16] was the first work to introduce a sparse network in self-supervised monocular depth estimation for laparoscopic scenes. However, there are two characteristics of laparoscopic scenes that make the task difficult.

First, the sparse key points in laparoscopic images present inherent challenges to self-supervised depth estimation. As shown in Fig. 1, the detected key points in laparoscopic images are sparse due to the smooth surfaces of the organ’s appearance, compared with autonomous driving or static scene datasets [10, 11]. The textureless regions decrease the photometric error even if there is a significant offset between the original pixels’ locations and projected pixels’ locations. As a second characteristic, the complex motions of the laparoscope make the depth and relative pose predictions challenging. Contrary to the motion range of cameras in other datasets, the laparoscope operates in a confined space, resulting in slight translations during surgical procedures. These minor translations produce less apparent differences between adjacent laparoscopic images, complicating the pixel matching carried out in a self-supervised learning strategy. In addition, complex rotations always accompany the laparoscope’s motions in three degrees of freedom, which differs from the motions of vehicles in autonomous driving datasets. This increases the difficulty of rotation predictions. Therefore, applying existing methods [4, 6, 7] for autonomous and static scene datasets to laparoscopic datasets did not lead to good performance. To overcome these challenges, Huang et al. [17] proposed self-supervised monocular depth estimation guided by 3D geometric consistency; however, their method was based on stereo images. Li et al. [18] and Li et al. [19] tried to leverage an auxiliary task and more complex view fusion to address the lack of features. However, these methods  [18, 19] greatly increased the number of parameters in the network, which necessitates greater computational and storage resources at training time. In addition, fewer network parameters and reduced training time can accelerate pre-trained model deployment and facilitate its adoption in clinical practice [20]. Accordingly, the motivation of this work is to maintain competitive performance in depth prediction for laparoscopic scenes while reducing model parameters.

Considering the characteristics of laparoscopic datasets and the limitations of previous methods [18, 19], we proposed several simple yet effective components rather than introducing auxiliary tasks with a large number of parameters. We propose a novel self-supervised monocular depth estimation method with a depth-pose loss function for laparoscopic datasets. Here, we replace the auxiliary tasks used in the previous method [18, 19] with several simple but effective components. Specifically, we extract feature maps and propose a joint depth-pose loss function to overcome the problem of the lack of features on RGB images and the challenging pose estimation for laparoscopic motion. This obtains more information from the existing depth estimation network instead of adding auxiliary tasks. Moreover, we introduce a self-attention block in the pose estimation network to focus on the informative regions.

This work mainly focuses on maintaining competitive performance in depth prediction for laparoscopic datasets while reducing model parameters. The contributions of this paper can be summarized as follows. (i) We use feature-metric loss with minimum operation to overcome the lack of features in smooth regions for laparoscopic images. (ii) We introduce a self-attention block in the pose estimation network to focus on the informative regions to predict relative poses from adjacent images. (iii) We calculate the difference of relative poses with Euler angles and translations as a pose loss function for pose estimation and build a depth-pose loss function for the entire learning strategy.

## Method

**Fig. 2:**
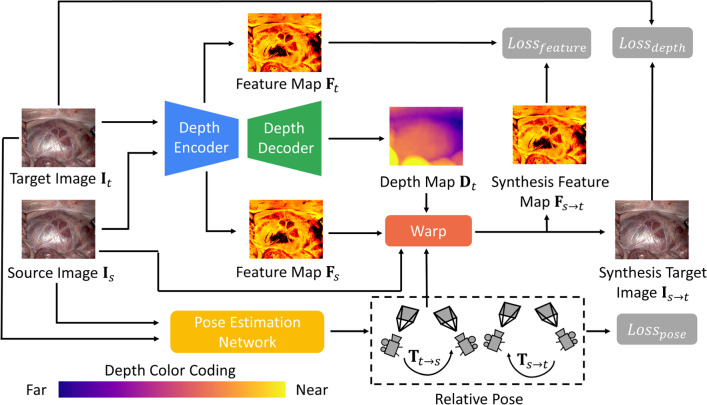
Flow of self-supervised learning strategy. The depth network generates the depth map ${\textbf {D}}_{t}$ and feature maps ${\textbf {F}}_{t}$ and ${\textbf {F}}_{s}$ from target image ${\textbf {I}}_{t}$ and source image ${\textbf {I}}_{s}$ by sharing the same encoder

### Self-supervised learning strategy

Following related work [4], we consider the self-supervised monocular depth estimation as a view-synthesis problem. As shown in Fig. 2, the learning strategy comprises two models: the depth estimation model and the pose estimation model. The inputs are target image ${\textbf {I}}_{t}$ from the view at time *t* and source images ${\textbf {I}}_{s}$ from another view at time *s*. Time *s* is either time $t-1$ or time $t+1$, representing the adjacent view at time *t*. We first predict depth map ${\textbf {D}}_{t}$ from ${\textbf {I}}_{t}$ by the depth estimation network. The pose estimation network’s inputs are ${\textbf {I}}_{t}$ and ${\textbf {I}}_{s}$, and it outputs the rigid transformation matrix as the relative pose.

Addressing the challenge of laparoscope rotations, we compute the average of two transformation matrices predicted by the pose estimation network. We feed ${\textbf {I}}_{t}$ and ${\textbf {I}}_{s}$ into the pose estimation network to predict transformation matrix $\textbf{T}_{t\rightarrow s}$. Then, we reverse the order of ${\textbf {I}}_{t}$ and ${\textbf {I}}_{s}$ as input images to estimate the transformation matrix $\textbf{T}_{s\rightarrow t}$. Here, $\textbf{T}_{t\rightarrow s}$ present the relative poses from ${\textbf {I}}_{t}$ and ${\textbf {I}}_{s}$, and $\textbf{T}_{s\rightarrow t}$ present the relative poses from ${\textbf {I}}_{s}$ and ${\textbf {I}}_{t}$. Then we calculate the mean of $\textbf{T}_{t\rightarrow s}$ and the inverse of $\textbf{T}_{s\rightarrow t}$ by

1 $$\begin{aligned} \overline{\textbf{T}}_{t\rightarrow s}=\frac{1}{2}({\textbf {T}}_{t\rightarrow s}+({\textbf {T}}_{s\rightarrow t})^{-1}), \end{aligned}$$

where $\overline{\textbf{T}}_{t\rightarrow s}$ denotes the averaged relative pose from the view of ${\textbf {I}}_{t}$ to the view of ${\textbf {I}}_{s}$. We obtain the 2D locations ${\textbf {p}}_{s}$ in ${\textbf {I}}_{s}$ based on ${\textbf {p}}_{t}$ in ${\textbf {I}}_{t}$ as

2 $$\begin{aligned} {\textbf {p}}_{s}={\textbf {K}}\overline{\textbf{T}}_{t\rightarrow s} {\textbf {D}}^{{\textbf {p}}_{t}} {\textbf {K}}^{-1}  {\textbf {p}}_{t}, \end{aligned}$$

where ${\textbf {D}}^{{\textbf {p}}_{t}}$ is the depth value at ${\textbf {p}}_{t}$ on the depth map ${\textbf {D}}$, and ${\textbf {K}}$ is a matrix as the intrinsic parameter of the laparoscope. The synthesized images ${\textbf {I}}_{s \rightarrow t}$ are generated by ${\textbf {I}}_{s\rightarrow t}^{{\textbf {p}}_{t}} \leftarrow {\textbf {I}}_{s}^{{\textbf {p}}_{s}}$. Time *s* belongs to the set including the adjacent times of time *t* as $s\in \left\{ t-1,t+1\right\}$. Following related work [4], we calculate the photometric error with the minimum operator as

3 $$\begin{aligned} \operatorname {E}\left( {\textbf {I}}_{t}^{\textbf{p}},{\textbf {I}}_{s\rightarrow t}^{\textbf{p}}\right) =\frac{\alpha }{2}\left( 1-\operatorname {SSIM}\left( {\textbf {I}}_{t}^{\textbf{p}},{\textbf {I}}_{s\rightarrow t}^{\textbf{p}}\right) \right) +\left( 1-\alpha \right) {\textbf {I}}_{t}^{\textbf{p}}-{\textbf {I}}_{s\rightarrow t}^{\textbf{p}}, \end{aligned}$$

4 $$\begin{aligned} L_m= \frac{1}{N}\sum _{\textbf{p} \in \textbf{H}}\min _{s}\operatorname {E}\left( {\textbf {I}}_{t}^{\textbf{p}}, {\textbf {I}}_{s\rightarrow t}^{\textbf{p}}\right) , \end{aligned}$$

where structured similarity (SSIM) [21] adopts $\alpha$ at 0.85, followed by related work [4]. $\textbf{p}$ is the 2D location of pixels. ${\textbf {I}}_{t}^{\textbf{p}}$ represents the value at $\textbf{p}$ on the target image ${\textbf {I}}_{t}$, and ${\textbf {I}}_{s\rightarrow t}^{\textbf{p}}$ represents the value at $\textbf{p}$ on the synthesized image ${\textbf {I}}_{s\rightarrow t}$. $\textbf{H}$ is a set including all the pixels’ 2D coordinates, and *N* is the number of pixels.

**Fig. 3:**
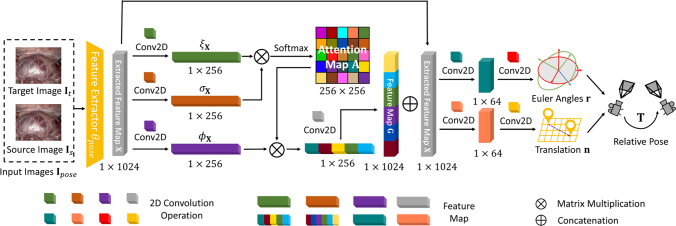
Architecture of pose estimation network guided by self-attention mechanism. The network has three main components: a feature extractor, a self-attention block, and two branches for outputs. The inputs are the target image and source image. The outputs include Euler angles and translations that are converted into transformation matrices as relative poses

### Attention-guided pose estimation network

Since the laparoscope makes complex motions during surgery, pose estimation in the self-supervised learning strategy becomes more challenging. The minor translations caused by the limited space result in less noticeable changes between target image ${\textbf {I}}_{t}$ and source image ${\textbf {I}}_{s}$. This renders the differences less apparent between ${\textbf {I}}_{t}$ and ${\textbf {I}}_{s}$, making the pose estimation more challenging. Therefore, we introduce the self-attention mechanism to the pose estimation network to focus on the informative regions of the input images illustrated in Fig. 3.

Following the classical self-attention mechanism [22], we represent the input of the pose estimation network as ${\textbf {I}}_{pose}$, which includes target image ${\textbf {I}}_{t}$ and source image ${\textbf {I}}_{s}$. First, the feature map is extracted from the input ${\textbf {I}}_{pose}$ by the pose estimation network’s encoder module $\theta _{pose}$ as ${\textbf {X}}=\theta _{pose}({\textbf {I}}_{pose})$. Then we utilize the self-attention block to reform ${\textbf {X}}$ as the query, key and value results by

5 $$\begin{aligned} \begin{aligned}&\xi _{{\textbf {X}}}=C_{\xi }({\textbf {X}}), \\&\delta _{{\textbf {X}}}=C_{\delta }({\textbf {X}}), \\&\phi _{{\textbf {X}}}=C_{\phi }({\textbf {X}}), \end{aligned} \end{aligned}$$

where $C_{\xi }$, $C_{\delta }$ and $C_{\phi }$ are 2D convolution operations with $3\times 3$ kernels. $\xi _{{\textbf {X}}}$, $\delta _{{\textbf {X}}}$ and $\phi _{{\textbf {X}}}$ are obtained query, key and value results. Then $\xi _{{\textbf {X}}}$ and $\delta _{{\textbf {X}}}$ are combined to generate the attention map ${\textbf {A}}$ by softmax as

6 $$\begin{aligned} {\textbf {A}}=S({\xi _{{\textbf {X}}}}^\textrm{T}\delta _{{\textbf {X}}}), \end{aligned}$$

where *S* is the nonlinear activation layer softmax. The attention map ${\textbf {A}}$, the value results $\phi _{{\textbf {X}}}$, and feature map ${\textbf {X}}$ contribute to the final feature maps as

7 $$\begin{aligned} {\textbf {G}} = C({\textbf {X}}+C(\phi _{{\textbf {X}}}  {\textbf {A}})), \end{aligned}$$

where *C* is 2D convolution operation with $3\times 3$ kernels. ${\textbf {G}}$ is the final feature map. As shown in Fig. 3, we obtain two final feature maps and output the Euler angles and translations, which are converted to the relative poses for the back-projection used in Eq. 2.

### Joint depth-pose loss function

Laparoscopic images present extensive textureless regions. This can cause misjudgment of the photometric error, making it appear smaller even when the predicted corresponding locations between ${\textbf {I}}_{t}$ and ${\textbf {I}}_{s}$ are not matched correctly. Similar to related work [23], we extract feature maps ${\textbf {F}}_{t}$ and ${\textbf {F}}_{s}$ from ${\textbf {I}}_{t}$ and ${\textbf {I}}_{s}$ with the depth estimation encoder module as shown in Fig. 2. To enforce feature consistency, we first use Eq. 2 to obtain the corresponding 2D locations ${\textbf {p}}_{t}^{f}$ and ${\textbf {p}}_{s}^{f}$ in ${\textbf {F}}_{t}$ and ${\textbf {F}}_{s}$. Then we synthesize feature map ${\textbf {F}}_{s\rightarrow t}$ based on ${\textbf {F}}_{s}$ by ${\textbf {F}}_{s\rightarrow t}^{{\textbf {p}}_{t}^{f}} \leftarrow {\textbf {F}}_{s}^{{\textbf {p}}_{s}^{f}}$. We calculate and select the minimum difference of the extracted feature map ${\textbf {F}}_{s}$ and synthesized feature map ${\textbf {F}}_{s\rightarrow t}$ as a feature-metric loss by

8 $$\begin{aligned} L_{f}= \frac{1}{N^{f}}\sum _{\textbf{p}^{f} \in \textbf{H}^{f}}\min _{s}\frac{\left| {\textbf {F}}_{t}^{\textbf{p}^{f}}-{\textbf {F}}_{s\rightarrow t}^{\textbf{p}^{f}}\right| }{{\textbf {F}}_{t}^{\textbf{p}^{f}}+{\textbf {F}}_{s\rightarrow t}^{\textbf{p}^{f}}}, \end{aligned}$$

where $\textbf{p}^{f}$ is the 2D location of pixels in a feature map. ${\textbf {F}}_{t}^{\textbf{p}^{f}}$ represents the value at $\textbf{p}^{f}$ on feature map ${\textbf {F}}_{t}$, and ${\textbf {F}}_{s\rightarrow t}^{\textbf{p}^{f}}$ represents the value at $\textbf{p}^{f}$ on the synthesized feature map ${\textbf {F}}_{s\rightarrow t}$. $\textbf{H}^{f}$ is a set including all of the pixels’ 2D coordinates in a feature map. $N^{f}$ is the number of pixels in a feature map. And time *s* belongs to the set as $s\in \left\{ t-1,t+1\right\}$. Furthermore, we exchange the channel positions of the target and source images in the input to predict the relative poses as $\textbf{T}_{t\rightarrow s}$ and $\textbf{T}_{s\rightarrow t}$ [18]. To ensure that the estimated poses satisfy the orthogonality constraint, we obtained the translations and rotations from $\textbf{T}_{t\rightarrow s}$ and $(\textbf{T}_{s\rightarrow t})^{-1}$ and calculated the difference between the obtained translations and rotations as

9 $$\begin{aligned} L_{r} = \left\| {\textbf {r}}_{t\rightarrow s}-\tilde{{\textbf {r}}}_{t\rightarrow s}\right\| _{1}, \end{aligned}$$

and

10 $$\begin{aligned} L_{n} = \left\| {\textbf {n}}_{t\rightarrow s}-\tilde{{\textbf {n}}}_{t\rightarrow s}\right\| _{1}, \end{aligned}$$

where $\left\| \cdot \right\| _{1}$ is the L1-norm operator. We use Euler angles to represent the rotations. ${\textbf {r}}_{t\rightarrow s}$ and ${\textbf {n}}_{t\rightarrow s}$ refer to Euler angles and translations of the predicted relative pose $\textbf{T}_{t\rightarrow s}$. Similarly, $\tilde{{\textbf {r}}}_{t\rightarrow s}$ and $\tilde{{\textbf {n}}}_{t\rightarrow s}$ represent the Euler angle and translation for the inverse of the predicted relative pose $(\textbf{T}_{s\rightarrow t})^{-1}$.

The entire pose loss function is a combination formed as $L_{p}=L_{r}+L_{n}$. The depth loss function is based on $L_{m}$ and $L_{f}$ as $L_{d}=L_{m}+\mu L_{f}$. The joint depth-pose loss function is defined by

11 $$\begin{aligned} L_{total} = L_{d}+\lambda L_{p}+\omega L_{s}, \end{aligned}$$

where $\mu$, $\lambda$, and $\omega$ are the weights for each component in the total loss function and $\mathcal {L}_{s}$ is the smoothness term in previous methods [4, 6].

**Table 1:** Quantitative comparison for depth estimation on SCARED with the number of parameters of each method at training time

Method	Abs Rel $\downarrow$	Sq Rel $\downarrow$	RMSE $\downarrow$	RMSE log $\downarrow$	$\gamma <1.25$ $\uparrow$	Params. $\downarrow$
Monodepth2 [4]	$0.081_{\pm 0.001}$	$0.953_{\pm 0.014}$	$8.029_{\pm 0.072}$	$0.105_{\pm 0.001}$	$0.936_{\pm 0.002}$	27.856 M
HRDepth [6]	$0.081_{\pm 0.002}$	$0.951_{\pm 0.055}$	$8.036_{\pm 0.249}$	$0.106_{\pm 0.002}$	$0.935_{\pm 0.005}$	27.627 M
Manydepth [24]	$0.079_{\pm 0.001}$	$0.959_{\pm 0.018}$	$7.754_{\pm 0.111}$	$0.103_{\pm 0.001}$	$0.937_{\pm 0.002}$	44.255 M
DIFFNet [25]	$0.086_{\pm 0.004}$	$1.059_{\pm 0.104}$	$8.286_{\pm 0.336}$	$0.110_{\pm 0.004}$	$0.929_{\pm 0.009}$	**23.883 M**
MonoViT [26]	$0.077_{\pm 0.002}$	$0.886_{\pm 0.037}$	$7.639_{\pm 0.157}$	$0.101_{\pm 0.002}$	$0.946_{\pm 0.003}$	40.884 M
BRNet [27]	$0.086_{\pm 0.001}$	$1.065_{\pm 0.019}$	$8.177_{\pm 0.141}$	$0.110_{\pm 0.001}$	$0.924_{\pm 0.002}$	37.986 M
GasMono [7]	$0.080_{\pm 0.000}$	$1.035_{\pm 0.039}$	$8.241_{\pm 0.111}$	$0.106_{\pm 0.000}$	$0.939_{\pm 0.000}$	28.531 M
Endo-SLAM [28]	$0.074_{\pm 0.001}$	$0.856_{\pm 0.001}$	$7.296_{\pm 0.028}$	$0.096_{\pm 0.001}$	$0.935_{\pm 0.001}$	27.854 M
SCDepth [16]	$0.070_{\pm 0.002}$	$0.754_{\pm 0.028}$	$6.986_{\pm 0.122}$	$0.093_{\pm 0.002}$	$0.949_{\pm 0.003}$	54.047 M
AF-SfMLearner [29]	$0.070_{\pm 0.002}$	$0.743_{\pm 0.057}$	$6.722_{\pm 0.109}$	$0.091_{\pm 0.003}$	$0.947_{\pm 0.008}$	57.566 M
GCDepthL [18]	$0.071_{\pm 0.001}$	$0.802_{\pm 0.008}$	$7.101_{\pm 0.029}$	$0.094_{\pm 0.001}$	$0.940_{\pm 0.001}$	42.702 M
MGMNet [19]	$\mathbf {0.066_{\pm 0.000}}$	$\mathbf {0.660_{\pm 0.006}}$	$\mathbf {6.352_{\pm 0.016}}$	$\mathbf {0.085_{\pm 0.000}}$	$\mathbf {0.958_{\pm 0.001}}$	44.257 M
Baseline	$0.083_{\pm 0.001}$	$0.929_{\pm 0.006}$	$7.872_{\pm 0.027}$	$0.102_{\pm 0.001}$	$0.941_{\pm 0.001}$	27.856 M
Ours	$\underline{0.068_{\pm 0.001}}$	$\underline{0.713_{\pm 0.008}}$	$\underline{6.679_{\pm 0.011}}$	$\underline{0.089_{\pm 0.001}}$	$\underline{0.954_{\pm 0.002}}$	36.036 M

M represents the number of parameters (Params.) in millions. Best performance is denoted using boldface. Second-best performance is denoted using underlines

## Experiments and results

### Datasets

We conducted all experiments using SCARED [13] and Hamlyn datasets processed by the method of a previous work [14]. SCARED gathered datasets on the abdominal anatomy of fresh porcine cadavers, comprising 9 different scenes in 35 videos. Hamlyn datasets consist of 22 phantom heart model videos, and they were annotated using the Efficient Large-Scale Stereo Matching algorithm [14]. The depth values in the ground truth were recorded in millimeters.

**Fig. 4:**
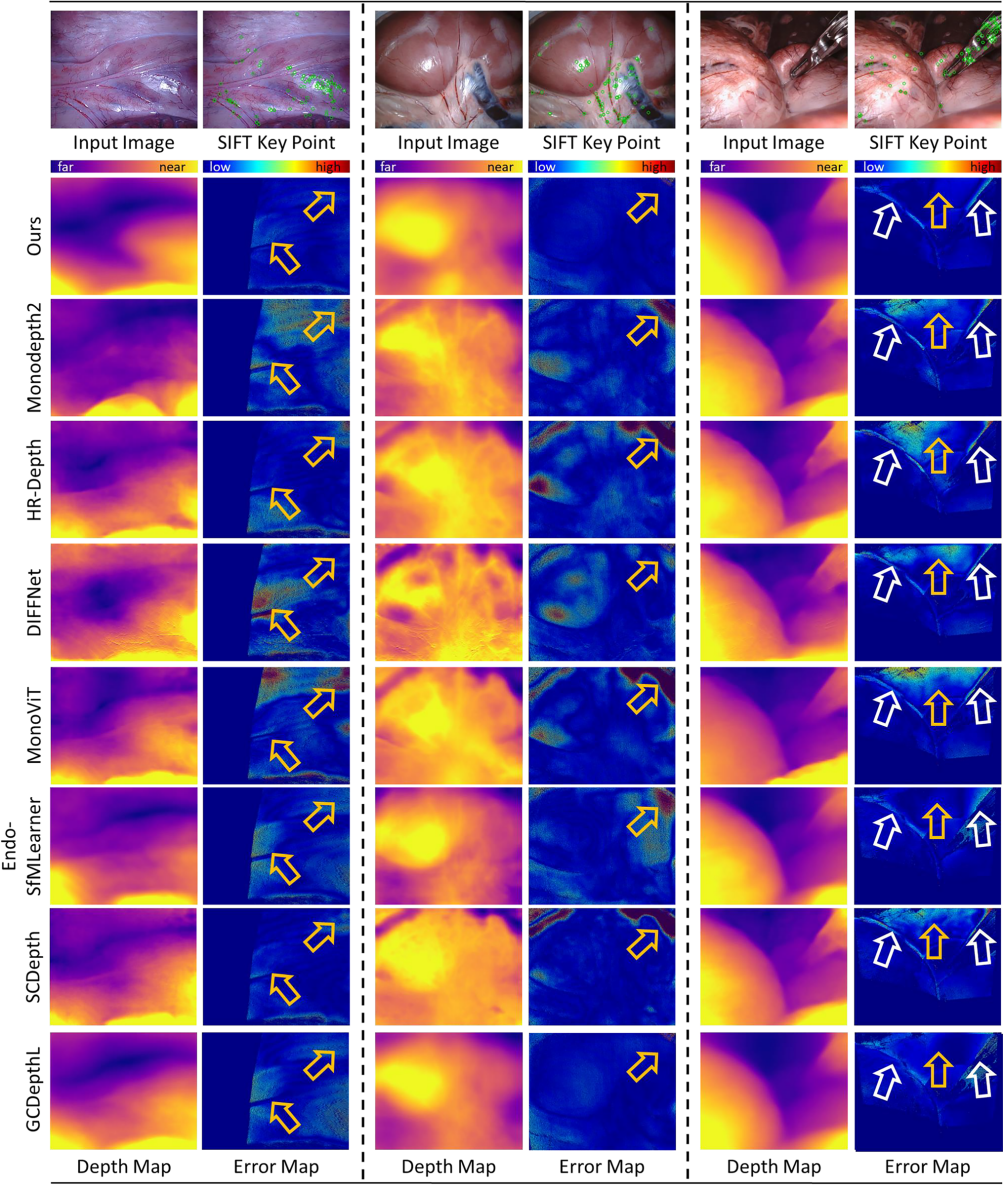
Examples of predicted depth maps, key point detection results obtained by SIFT [15] and error maps calculated by the absolute relative error (Abs Rel) of the predicted depth maps and ground-truth on SCARED. The key points extracted by SIFT [15] are shown with less texture in SCARED. Yellow arrows indicate regions where the proposed method outperformed previous methods. White arrows highlight the edges of surgical tools where both the proposed method and previous methods performed poorly

We split each dataset into training and testing sets in a 10:1 ratio according to the video sequence of each scene, thus ensuring consecutive frames in both datasets. The experiments on SCARED were executed on 23,687 and 2,405 frames for training and testing. Likewise, the experiments on Hamlyn datasets were split into 21,090 and 2,014 frames for training and testing. Due to constraints imposed by computational resources, we downsampled images to 320 $\times$ 256 pixels for both datasets.

### Implementation details

We developed our model using the PyTorch library [30] and trained the networks over 30 epochs employing Adam [31] with $\beta _{1}=0.9$ and $\beta _{2}=0.99$. The learning rate was initialized at $10^{-4}$ and subsequently scaled by a factor of 0.1 after 15 epochs. We used a training batch size of 12. The parameters $\mu$ and $\lambda$ were established at 0.1, and $\omega$ was set to 0.001. Moreover, we constrained the cap of predicted depth to 150 mm.

We adopted ResNet-18 as the encoder module for depth estimation and pose estimation networks. We employed the same decoder modules as outlined in Monodepth2 [4]. The feature maps are extracted from the first residual block of ResNet-18. The height and width of feature maps are half those of the original image, with 64 channels. The experiment was conducted on a single NVIDIA Quadro RTX 8000 GPU. The proposed method took about 8 h for training on the SCARED and Hamlyn datasets, respectively.

**Table 2:** Quantitative comparison for depth estimation on Hamlyn datasets with the number of parameters of each method at training time

Method	Abs Rel $\downarrow$	Sq Rel $\downarrow$	RMSE $\downarrow$	RMSE log $\downarrow$	$\gamma <1.25$ $\uparrow$	Params. $\downarrow$
Monodepth2 [4]	$0.176_{\pm 0.002}$	$4.422_{\pm 0.140}$	$16.406_{\pm 0.242}$	$0.212_{\pm 0.002}$	$0.754_{\pm 0.003}$	27.856 M
HRDepth [6]	$0.171_{\pm 0.001}$	$4.028_{\pm 0.090}$	$15.627_{\pm 0.130}$	$0.206_{\pm 0.001}$	$0.755_{\pm 0.001}$	27.627 M
Manydepth [24]	$0.166_{\pm 0.001}$	$3.881_{\pm 0.090}$	$15.477_{\pm 0.130}$	$0.202_{\pm 0.001}$	$0.763_{\pm 0.001}$	44.255 M
DIFFNet [25]	$0.168_{\pm 0.005}$	$3.898_{\pm 0.179}$	$15.409_{\pm 0.409}$	$0.204_{\pm 0.005}$	$0.759_{\pm 0.011}$	**23.883 M**
MonoViT [26]	$0.175_{\pm 0.004}$	$4.413_{\pm 0.164}$	$16.254_{\pm 0.388}$	$0.213_{\pm 0.005}$	$0.752_{\pm 0.007}$	40.884 M
BRNet [27]	$0.163_{\pm 0.003}$	$3.652_{\pm 0.164}$	$15.013_{\pm 0.242}$	$0.198_{\pm 0.003}$	$0.768_{\pm 0.008}$	37.986 M
GasMono [7]	$0.186_{\pm 0.006}$	$4.305_{\pm 0.238}$	$15.932_{\pm 0.458}$	$0.226_{\pm 0.006}$	$0.718_{\pm 0.013}$	28.531 M
Endo-SLAM [28]	$0.181_{\pm 0.001}$	$3.941_{\pm 0.001}$	$16.229_{\pm 0.008}$	$0.215_{\pm 0.001}$	$0.704_{\pm 0.001}$	27.854 M
SCDepth [16]	$0.184_{\pm 0.002}$	$4.574_{\pm 0.192}$	$16.961_{\pm 0.248}$	$0.222_{\pm 0.002}$	$0.724_{\pm 0.004}$	54.047 M
AF-SfMLearner [29]	$0.166_{\pm 0.002}$	$3.881_{\pm 0.057}$	$15.477_{\pm 0.109}$	$0.202_{\pm 0.003}$	$0.763_{\pm 0.008}$	57.566 M
GCDepthL [18]	$0.162_{\pm 0.001}$	$\underline{3.302_{\pm 0.035}}$	$14.762_{\pm 0.079}$	$0.196_{\pm 0.001}$	$0.749_{\pm 0.001}$	42.702 M
MGMNet [19]	$\underline{0.159_{\pm 0.004}}$	$3.340_{\pm 0.179}$	$\underline{14.553_{\pm 0.325}}$	$\underline{0.193_{\pm 0.004}}$	$\underline{0.770_{\pm 0.007}}$	44.257 M
Baseline	$0.179_{\pm 0.005}$	$4.526_{\pm 0.223}$	$16.507_{\pm 0.382}$	$0.215_{\pm 0.005}$	$0.744_{\pm 0.008}$	27.856 M
Ours	$\mathbf {0.154_{\pm 0.001}}$	$\mathbf {3.037_{\pm 0.012}}$	$\mathbf {13.859_{\pm 0.050}}$	$\mathbf {0.185_{\pm 0.001}}$	$0.768_{\pm 0.001}$	36.036 M

M represents the number of parameters (Params.) in millions. Best performance is denoted using boldface. Second-best performance is denoted as underlines

**Fig. 5:**
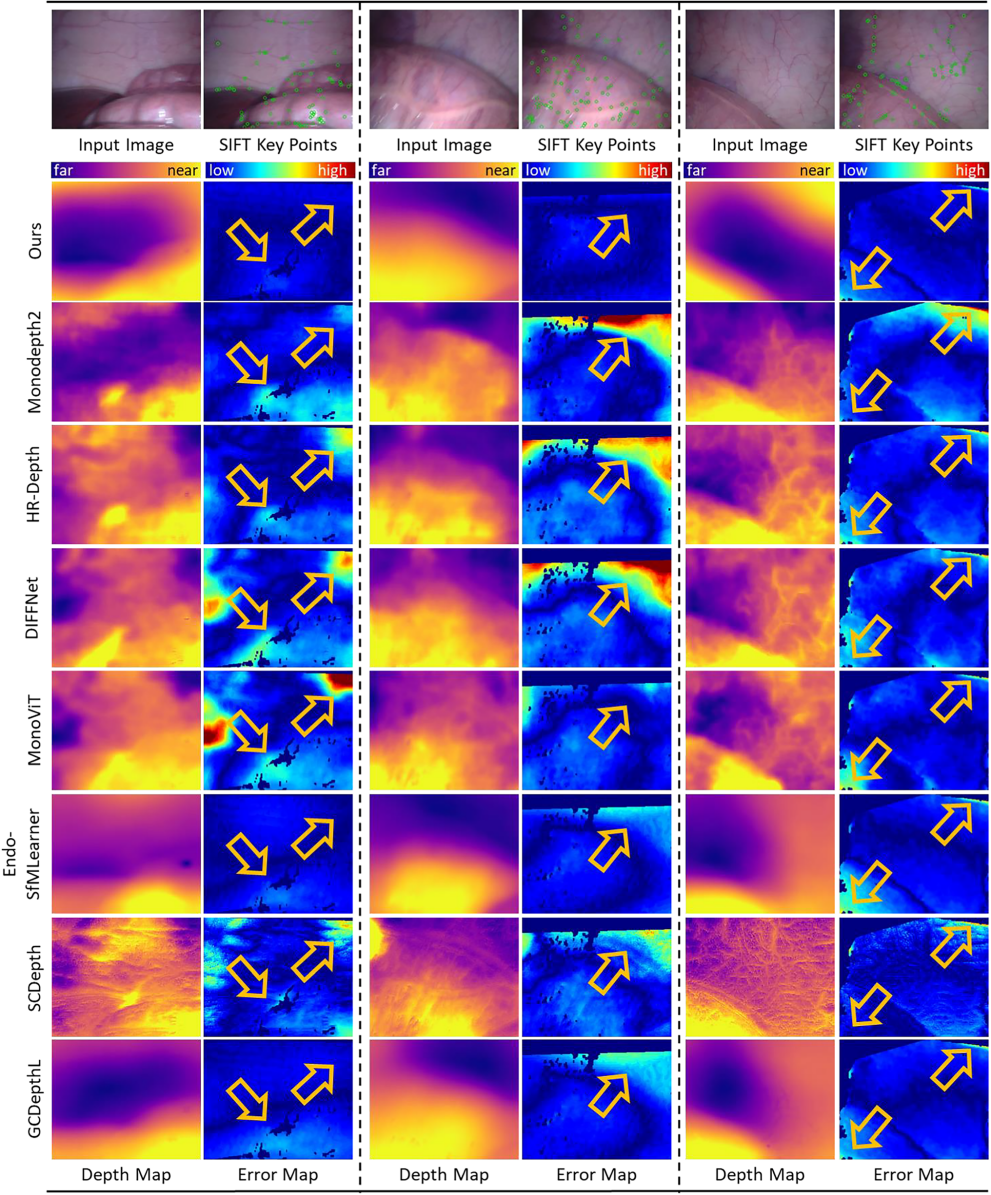
Examples of predicted depth maps, key point detection results obtained by SIFT [15] and error maps calculated by the absolute relative error (Abs Rel) of the predicted depth maps and ground-truth on Hamlyn datasets. The key points extracted by SIFT [15] are shown the less texture in Hamlyn datasets. The key points are shown the less texture in Hamlyn datasets. Yellow arrows indicate regions where the proposed method outperformed previous methods

### Comparison results

We selected methods that have been applied not only to laparoscopic scenes [16, 18, 19, 28, 29] but also to autonomous driving datasets and static scene datasets [4, 6, 7, 24–27]. All of the methods used ResNet-18 as a backbone for a fair comparison. Since the focus of this work is to improve the performance of self-supervised monocular depth estimation on laparoscopic images, we primarily compared the performance of the proposed method with those of existing methods [4, 6, 7, 16, 18, 19, 24–29] for depth estimation on two datasets. We retrained these existing methods [4, 6, 7, 16, 18, 19, 24–29] on the SCARED and Hamlyn datasets, sharing the same hyperparameters. We compared the results of the proposed method and existing methods on depth estimation quantitatively and qualitatively. For all of the predicted depth maps, we employed median scaling of the predicted depth values and ground truth to solve the scale problem, as in the previous methods [4] by

12 $$\begin{aligned} {\textbf {D}}_{scaled} = (M({\textbf {D}}^{*}) / M({\textbf {D}})){\textbf {D}}, \end{aligned}$$

where ${\textbf {D}}$ represents predicted depth map, ${\textbf {D}}^{*}$ represents ground-truth, and the *M* operator obtained the median values of ${\textbf {D}}^{*}$ and ${\textbf {D}}$. ${\textbf {D}}_{scaled}$ represents scaled depth maps. ${\textbf {D}}_{scaled}$ and ${\textbf {D}}^{*}$ were used to evaluate the performance of each method.

**Fig. 6:**
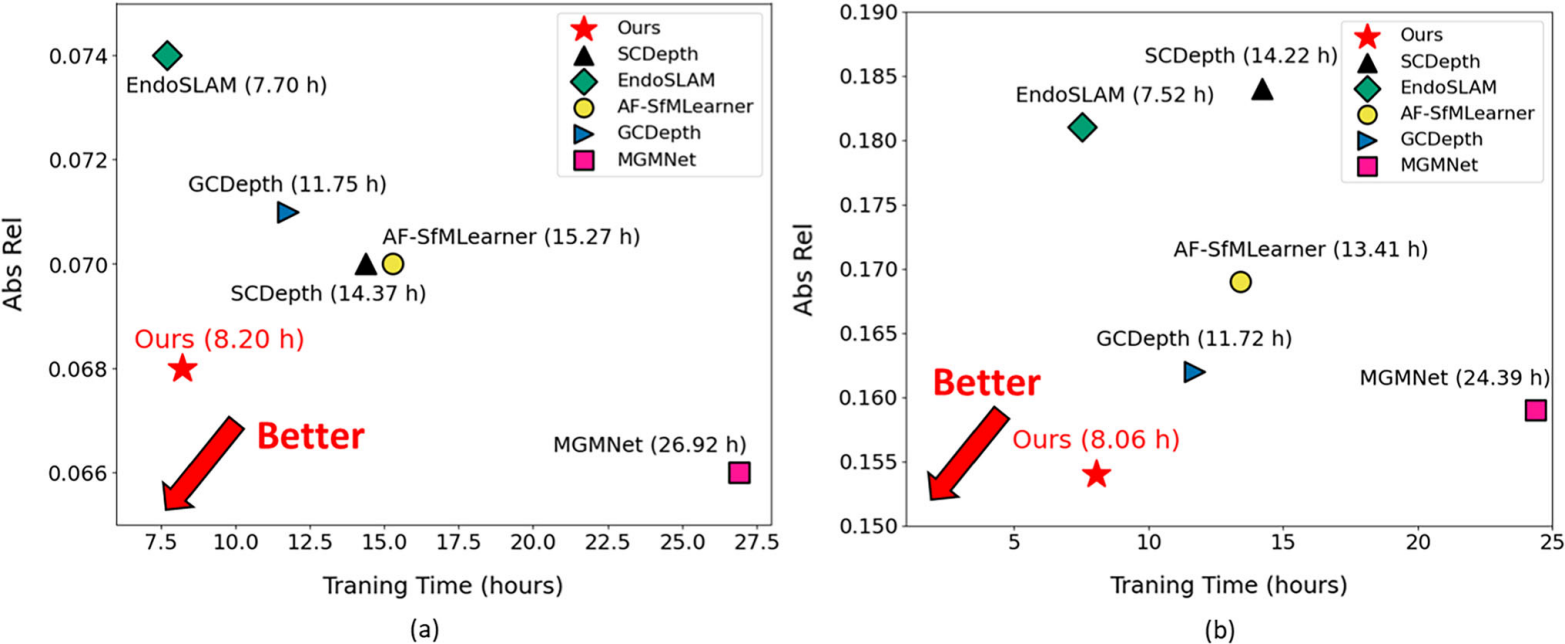
Comparison of absolute relative error (Abs Rel) and training time on different methods designed for laparoscopic datasets [16, 18, 19, 28, 29]. **a** Abs Rel on SCARED with training time; **b** Abs Rel on Hamlyn datasets with training time

For quantitative comparison, we adopted the five widely used criteria [4] to evaluate the results of the proposed method and other methods on SCARED and Hamlyn datasets. All experiments were repeated three times based on different seeds. The means and standard deviations of the results for these two datasets are listed in Tables 1 and 2. For qualitative comparison, we outputted the predicted depth maps and corresponding error maps from each method. The error maps were calculated by the absolute relative error (Abs Rel) between the predicted depth maps and ground-truth. In addition, we extracted the key points of original images by SIFT [15], as shown in Figs. 4 and 5. Moreover, we provided the total number of neural network parameters at training time for each method in Tables 1 and 2. We also compared Abs Rel and training time on different methods as shown in Fig. 6.

### Ablation study

To better analyze the contributions of each component in the proposed method, we evaluated variants of the method to complete an ablation study. Each component’s contribution to the proposed method was separately evaluated based on the depth evaluation. Table 3 shows the performance of the three different proposed components: self-attention block (SB), feature-metric loss function $L_{f}$, and pose loss function $L_{p}$. In this table, we list all variants of the proposed method based on these three components. We assign ID numbers to each result for performance analysis.

**Table 3:** Evaluation of variants of the proposed model on SCARED

ID	SB	$L_{f}$	$L_{p}$	Abs Rel $\downarrow$	Sq Rel $\downarrow$	RMSE $\downarrow$	RMSE log $\downarrow$	$\gamma <1.25$ $\uparrow$
1				$0.083_{\pm 0.001}$	$0.929_{\pm 0.006}$	$7.872_{\pm 0.027}$	$0.102_{\pm 0.001}$	$0.941_{\pm 0.001}$
2	✓			$0.079_{\pm 0.002}$	$0.893_{\pm 0.006}$	$7.646_{\pm 0.027}$	$0.102_{\pm 0.001}$	$0.943_{\pm 0.001}$
3		✓		$0.080_{\pm 0.001}$	$0.896_{\pm 0.006}$	$7.687_{\pm 0.004}$	$0.102_{\pm 0.001}$	$0.940_{\pm 0.002}$
4			✓	$0.079_{\pm 0.002}$	$0.893_{\pm 0.014}$	$7.646_{\pm 0.083}$	$0.102_{\pm 0.003}$	$0.943_{\pm 0.003}$
5	✓	✓		$0.072_{\pm 0.001}$	$0.808_{\pm 0.009}$	$7.119_{\pm 0.015}$	$0.094_{\pm 0.001}$	$0.944_{\pm 0.003}$
6	✓		✓	$\underline{0.071_{\pm 0.001}}$	$0.778_{\pm 0.019}$	$7.019_{\pm 0.039}$	$0.093_{\pm 0.001}$	$0.947_{\pm 0.002}$
7		✓	✓	$\underline{0.071_{\pm 0.001}}$	$\underline{0.759_{\pm 0.008}}$	$\underline{6.913_{\pm 0.053}}$	$\underline{0.092_{\pm 0.001}}$	$\underline{0.950_{\pm 0.001}}$
8	✓	✓	✓	$\mathbf {0.068_{\pm 0.001}}$	$\mathbf {0.713_{\pm 0.008}}$	$\mathbf {6.679_{\pm 0.011}}$	$\mathbf {0.089_{\pm 0.001}}$	$\mathbf {0.954_{\pm 0.002}}$

SB: self-attention block; $L_{f}$: feature-metric loss function; $L_{p}$: pose loss function. Best performance is denoted using boldface. Second-best performance is denoted using underlines

## Discussion

Laparoscopic images involve various scenes and smooth surfaces of organs, which causes the depth estimation performance not well on each scene.

As shown in Tables 1 and 2, the experimental results show that the existing methods for the autonomous driving and static scene datasets [4, 6, 7, 24–27] performed worse than the proposed method on SCARED and Hamlyn datasets. Moreover, the proposed method provides improved performance compared with the previous models [4, 6, 7, 16, 18, 19, 24–29] for SCARED and Hamlyn datasets. Furthermore, the small variance across three experimental repetitions demonstrates our method’s quantitative robustness. However, the proposed method performed slightly worse than MGMNet [19]. MGMNet [19] incorporated two additional neural networks for different tasks (multi-view depth estimation and offset prediction). It leveraged their results to guide monocular depth estimation. This enabled MGMNet [19] to capture richer feature representations from different views and realize deformable patch-matching, making it particularly effective in various and challenging scenarios like SCARED. In comparison, as shown in Tables 1 and 2, our work reduces the number of parameters by 18.57%, while only incurring a 3.03% decrease in Abs Rel performance for SCARED compared to MGMNet [19]. Moreover, the proposed method even showed a 3.14% improvement on Abs Rel for Hamlyn datasets compared to MGMNet [19]. As shown in Fig. 6, the proposed method also had a 69.54% and 66.95% reduction in training time for SCARED and Hamlyn datasets compared to MGMNet [19]. And, the proposed method reduces the number of parameters of networks for SCARED and Hamlyn datasets by 33.32% and 16.00%, but it improves the performance on both datasets significantly compared to SCDepth [16] and GCDepthL [18]. This shows that the proposed method performs well and strikes an efficient balance between computational efficiency and performance.

Furthermore, we show the qualitative performance of three different laparoscopic scenes. According to the first two columns of Fig. 4, the proposed model performs smoothly and robustly for each scene according to the predicted depth maps and error maps. In the final column of Fig. 4, several previous methods [18, 28] also performed smooth and clear depth maps. However, compared to the previous methods, the proposed method still has obvious but low errors in some regions. Consequently, despite the low error in some regions, these regions still look similar and smooth in the predicted depth map. The yellow arrows in Figs. 4 and 5 showed the regions where the depth maps predicted by the proposed method exhibit lower errors. The limitation of the proposed method is its tendency to produce larger errors in regions with sharp depth changes, particularly along the edges of surgical instruments, as shown by the white arrows in Fig. 4. As shown in Fig. 5, the proposed method also generated smooth depth maps with low error in Hamlyn datasets.

According to the performance with the ID numbers in Table 3, each component contributes to the proposed method’s performance (ID 2, ID 3, and ID 4). In particular, the self-attention-guided pose estimation network with the pose loss function enhanced performance compared to a single proposed component for pose estimation (ID 2, ID 4, and ID 6). The feature-metric loss function with any proposed component for pose estimation improved the results significantly (ID 3, ID 5, and ID 7). Furthermore, the total proposed method showed a noticeable improvement when combining all components (ID 1 and ID 8).

## Conclusion

In this work, we first analyzed the characteristics of the laparoscopic images and the motions of laparoscopes. We proposed a novel self-supervised monocular depth estimation method with a self-attention block. We also built a joint depth-pose loss function including a feature-metric loss function based on the extracted feature maps and a novel pose loss function. The quantitative experimental results show that our proposed model outperforms the existing models and provides robust performance. Furthermore, the qualitative results show that the proposed method predicts smooth and robust depth maps in different scenes with lower error compared to other methods. In future work, we will consider the non-rigid transform problem of soft issues in real-time surgical scenes and attempt to leverage the 3D information and temporal information to improve depth estimation.

## References

[1] Takada C, Afifi A, Suzuki T, Nakaguchi T (2017) An enhanced hybrid tracking-mosaicking approach for surgical view expansion. In: 2017 39th annual international conference of the IEEE engineering in medicine and biology society (EMBC), pp 3692–3695 10.1109/EMBC.2017.8037659 29060700

[2] Vecchio R, MacFayden B, Palazzo F (2000) History of laparoscopic surgery. Panminerva Med 42(1):87–90 11019611

[3] Qian L, Zhang X, Deguet A, Kazanzides P (2019) ARAMIS: augmented reality assistance for minimally invasive surgery using a head-mounted display. In: Medical image computing and computer assisted intervention–MICCAI 2019: 22nd international conference, LNCS, vol 11768, pp 74–82

[4] Godard C, Mac Aodha O, Firman M, Brostow GJ (2019) Digging into self-supervised monocular depth estimation. In: Proceedings of the IEEE/CVF international conference on computer vision, pp 3828–3838

[5] Zhou T, Brown M, Snavely N, Lowe DG (2017) Unsupervised learning of depth and ego-motion from video. In: 2017 IEEE conference on computer vision and pattern recognition (CVPR), pp 6612–6619

[6] Lyu X, Liu L, Wang M, Kong X, Liu L, Liu Y, Chen X, Yuan Y (2021) HR-depth: high resolution self-supervised monocular depth estimation. In: Proceedings of the AAAI conference on artificial intelligence, vol 35, pp 2294–2301

[7] Zhao C, Poggi M, Tosi F, Zhou L, Sun Q, Tang Y, Mattoccia S (2023) GasMono: geometry-aided self-supervised monocular depth estimation for indoor scenes. In: Proceedings of the IEEE/CVF international conference on computer vision, pp 16209–16220

[8] Saunders K, Vogiatzis G, Manso LJ (2023) Self-supervised monocular depth estimation: let’s talk about the weather. In: Proceedings of the IEEE/CVF International Conference on Computer Vision, pp 8907–8917

[9] Wang R, Yu Z, Gao S (2023) Planedepth: self-supervised depth estimation via orthogonal planes. In: Proceedings of the IEEE/CVF conference on computer vision and pattern recognition, pp. 21425–21434

[10] Shotton J, Glocker B, Zach C, Izadi S, Criminisi A, Fitzgibbon A (2013) Scene coordinate regression forests for camera relocalization in RGB-D images. In: 2013 IEEE conference on computer vision and pattern recognition, pp 2930–2937

[11] Menze M, Geiger A (2015) Object scene flow for autonomous vehicles. In: 2015 IEEE conference on computer vision and pattern recognition (CVPR), pp 3061–3070

[12] Ye M, Johns E, Handa A, Zhang L, Pratt P, Yang G-Z (2017) Self-supervised siamese learning on stereo image pairs for depth estimation in robotic surgery. In: The Hamlyn symposium on medical robotics, p 27

[13] Allan M, Mcleod J, Wang C, Rosenthal JC, Hu Z, Gard N, Eisert P, Fu KX, Zeffiro T, Xia W et al (2021) Stereo correspondence and reconstruction of endoscopic data challenge. arXiv preprint arXiv:2101.01133

[14] Recasens D, Lamarca J, Fácil JM, Montiel J, Civera J (2021) Endo-depth-and-motion: reconstruction and tracking in endoscopic videos using depth networks and photometric constraints. IEEE Robot Autom Lett 6(4):7225–7232

[15] Lowe DG (2004) Distinctive image features from scale-invariant keypoints. Int J Comput Vis 60(2):91–110

[16] Li W, Hayashi Y, Oda M, Kitasaka T, Misawa K, Mori K (2022) Spatially variant biases considered self-supervised depth estimation based on laparoscopic videos. Comput Methods Biomech Biomed Eng Imaging Vis 10(3):274–282

[17] Huang B, Zheng J-Q, Nguyen A, Xu C, Gkouzionis I, Vyas K, Tuch D, Giannarou S, Elson DS (2022) Self-supervised depth estimation in laparoscopic image using 3D geometric consistency. In: Medical image computing and computer assisted intervention–MICCAI 2022: 25th international conference, LNCS, vol 13437, pp 13–22

[18] Li W, Hayashi Y, Oda M, Kitasaka T, Misawa K, Mori K (2022) Geometric constraints for self-supervised monocular depth estimation on laparoscopic images with dual-task consistency. In: Medical image computing and computer assisted intervention-MICCAI 2022: 25th international conference, Singapore, Sept 18–22, 2022, Proceedings, LNCS, vol. 13434, pp. 467–477

[19] Li W, Hayashi Y, Oda M, Kitasaka T, Misawa K, Mori K (2023) Multi-view guidance for self-supervised monocular depth estimation on laparoscopic images via spatio-temporal correspondence. In: Medical image computing and computer assisted intervention—MICCAI 2023: 26th international conference, LNCS, vol 14228, pp 429–439

[20] Murshed MS, Murphy C, Hou D, Khan N, Ananthanarayanan G, Hussain F (2021) Machine learning at the network edge: a survey. ACM Comput Surv (CSUR) 54(8):1–37

[21] Wang Z, Bovik AC, Sheikh HR, Simoncelli EP (2004) Image quality assessment: from error visibility to structural similarity. IEEE Trans Image Process 13(4):600–612 15376593 10.1109/tip.2003.819861

[22] Wang X, Girshick R, Gupta A, He K (2018) Non-local neural networks. In: 2018 IEEE/CVF conference on computer vision and pattern recognition, pp 7794–7803

[23] Shu C, Yu K, Duan Z, Yang K (2020) Feature-metric loss for self-supervised learning of depth and egomotion. In: Computer vision—ECCV 2020, pp 572–588

[24] Watson J, Mac Aodha O, Prisacariu V, Brostow G, Firman M (2021) The temporal opportunist: self-supervised multi-frame monocular depth. In: Proceedings of the IEEE/CVF conference on computer vision and pattern recognition, pp 1164–1174

[25] Zhou H, Greenwood D, Taylor S (2021) Self-supervised monocular depth estimation with internal feature fusion. In: British machine vision conference (BMVC)

[26] Zhao C, Zhang Y, Poggi M, Tosi F, Guo X, Zhu Z, Huang G, Tang Y, Mattoccia S (2022) MonoViT: self-supervised monocular depth estimation with a vision transformer. In: 2022 International conference on 3d vision (3DV), pp 668–678

[27] Han W, Yin J, Jin X, Dai X, Shen J (2022) BRNet: exploring comprehensive features for monocular depth estimation. In: Computer vision—ECCV 2022, pp 586–602

[28] Ozyoruk KB, Gokceler GI, Bobrow TL, Coskun G, Incetan K, Almalioglu Y, Mahmood F, Curto E, Perdigoto L, Oliveira M et al (2021) EndoSLAM dataset and an unsupervised monocular visual odometry and depth estimation approach for endoscopic videos. Med Image Anal 71:102058 33930829 10.1016/j.media.2021.102058

[29] Shao S, Pei Z, Chen W, Zhu W, Wu X, Sun D, Zhang B (2022) Self-supervised monocular depth and ego-motion estimation in endoscopy: appearance flow to the rescue. Med Image Anal 77:102338 35016079 10.1016/j.media.2021.102338

[30] Paszke A, Gross S, Chintala S, Chanan G, Yang E, DeVito Z, Lin Z, Desmaison A, Antiga L, Lerer A (2017) Automatic differentiation in pytorch. In: NIPS 2017 workshop on autodiff

[31] Kingma DP, Ba J (2015) Adam: a method for stochastic optimization. In: 3rd International conference on learning representations, ICLR 2015, San Diego, CA, USA, May 7–9, 2015, Conference track proceedings

